# Subpleural emphysema as a rare initial manifestation of an iatrogenic tracheal penetration: a case report

**DOI:** 10.1186/s40792-022-01556-w

**Published:** 2022-10-22

**Authors:** Minori Nakamura, Shuhei Iizuka, Toru Nakamura

**Affiliations:** Department of General Thoracic Surgery, Seirei Hamamatsu General Hospital, 2-12-12, Sumiyoshi, Naka-Ku, Hamamatsu-City, Shizuoka 430-8558 Japan

**Keywords:** Double-lumen tube, Subpleural emphysema, Iatrogenic, Tracheal injury

## Abstract

**Background:**

Iatrogenic tracheal injury is a rare but potentially morbid condition and often poses a diagnostic challenge due to its rarity and the lack of specific clinical findings. Because a delayed diagnosis is associated with a higher mortality, a prompt diagnosis is essential. We report a case of an iatrogenic tracheal injury detected by subpleural emphysema as a rare initial manifestation.

**Case presentation:**

A 75-year-old woman was diagnosed with stage IA2 right lung cancer. During the surgery, visceral subpleural emphysema developed along the lung surface up to the interlobar fissure followed by subcutaneous emphysema in the anterior neck. Suspecting a tracheal injury, we aborted the surgery. Fiberoptic bronchoscopy revealed a longitudinal laceration on the membranous part of the distal trachea without esophageal involvement, consistent with a level II injury. Conservative management was chosen and she had a successful recovery.

**Conclusions:**

Iatrogenic tracheal injury could initially manifest as visceral subpleural emphysema. Once subpleural emphysema is observed during surgery, a prompt diagnostic workup of the tracheal injury should be performed.

## Background

An iatrogenic tracheal injury is a rare but potentially morbid condition. It often poses a diagnostic challenge due to its rarity and the lack of specific clinical findings, and a delayed diagnosis is associated with a higher mortality [1].

Tracheobronchial injury due to a double-lumen tube (DLT) commonly involves the membranous portion of the left main bronchus and distal trachea and could manifest as an intraoperative air leak, subcutaneous emphysema, or respiratory impairment. We report a case of a tracheal injury during a DLT intubation detected by subpleural emphysema as a rare initial manifestation.

## Case presentation

**Fig. 1:**
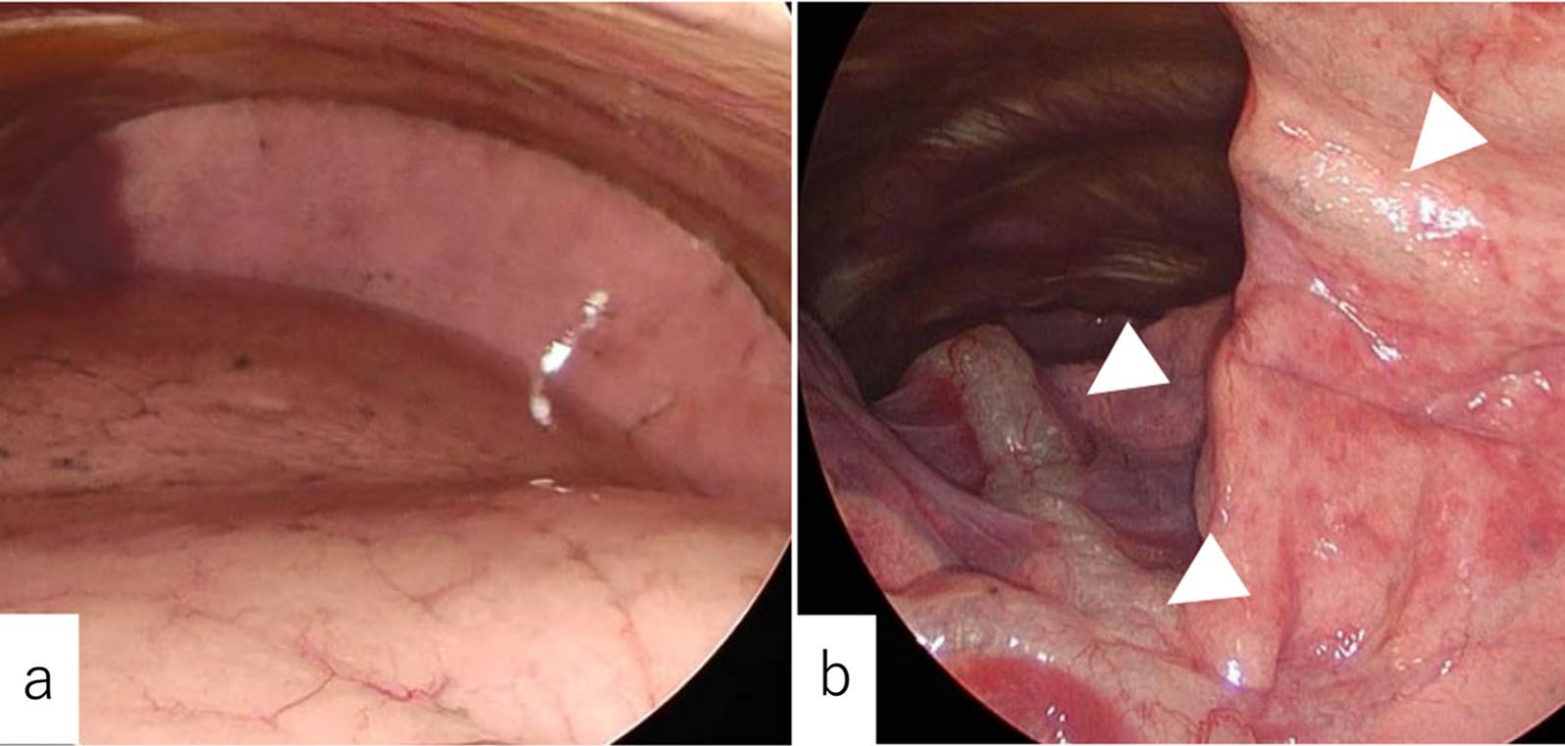
**a** The exploratory thoracoscopy revealed a normal lung appearance at the beginning of the surgery. **b** Subpleural emphysema (arrowheads) developed after fixing the position of the DLT

**Fig. 2:**
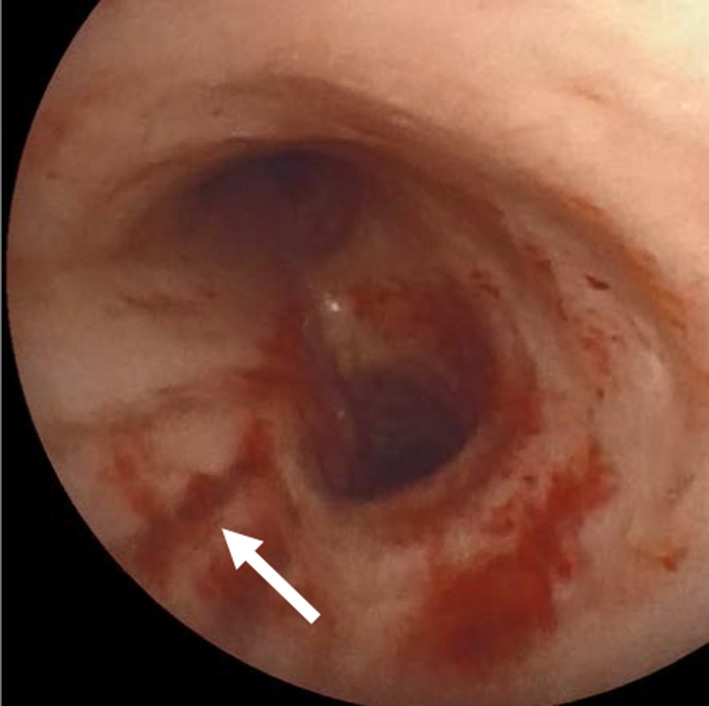
Fiberoptic bronchoscopy revealed a longitudinal laceration on the membranous part of the distal trachea (arrow)

A 75-year-old woman presented with a right pulmonary nodule. She was 151 cm tall, weighed 61 kg with a body mass index as 27.6. Her medical history included hypertension, hyperlipidemia, a fatty liver, and a left kidney stone. The pulmonary function test yielded normal results with a vital capacity of 2.55 L (118.6% predicted value) and forced expiratory volume over 1 s of 2.03 L (113.4% predicted value). A transbronchial biopsy revealed an adenocarcinoma, and she was diagnosed with stage IA2 right lung cancer. A right lower lobectomy was planned. Anesthesia was successfully induced with propofol, remifentanil, rocuronium bromide, and desflurane. A 37 French left-sided double-lumen tube (COOPDECH Endobronchial Blocker Tube, DAIKEN MEDICAL CO. LTD, Japan) was placed by an attending anesthesiologist during the initial attempt. Fiberoptic bronchoscopy verified the proper tube placement. With the patient in a left-lateral decubitus position, an exploratory thoracoscopy revealed a normal lung appearance (Fig. 1a). While placing the access port, she developed severe hypoxia with an oxygen saturation of 80% and elevated peak inspiratory pressure (PIP) of 25 cm H_2_O. The surgery was temporarily suspended and the bronchial tube tip was found to be placed too deep and obstructing the left upper lobe branch. Fixing the tube to the proper depth insertion improved the oxygenation with a normalized PIP. After achieving one-lung ventilation, a repeated thoracoscopy revealed subpleural emphysema along the lung surface up to the interlobar fissure (Fig. 1b) followed by subcutaneous emphysema in the anterior neck. Suspecting a tracheal injury, we aborted the surgery. The DLT was extubated and substituted with a laryngeal mask airway. Fiberoptic bronchoscopy revealed a longitudinal laceration on the membranous part of the distal trachea without an esophageal involvement (Fig. 2). It was consistent with a level II tracheal injury from the DLT intubation accompanied by subcutaneous emphysema [2]. Conservative management was chosen because of the limited extent of the superficial injury without any respiratory impairment.

**Fig. 3:**
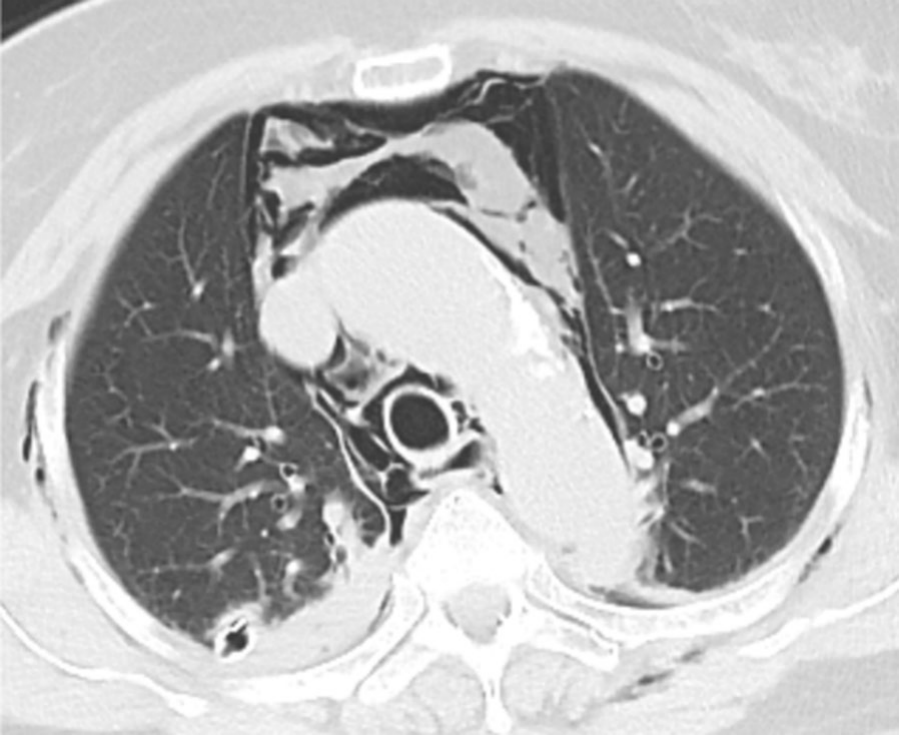
Postoperative CT revealed mediastinal and subcutaneous emphysema without any evident tracheal injury

The laryngeal mask airway was removed in the operating room to avoid any further deterioration of the tracheal wall by positive air way pressure. Postoperative computed tomography (CT) showed extensive mediastinal and subcutaneous emphysema without any tracheal injury noted (Fig. 3). That emphysema regressed spontaneously and she was discharged on the 2nd postoperative day.

## Discussion

The incidence of iatrogenic tracheal injury from a DLT is greater than among all other endotracheal intubation devices. The other risk factors for tracheobronchial injury in the present case included an older age than 65 years, female gender, short body height, and the use of a stylet [3]. Without any other specific factors such as bronchitis or steroid use, the cause of the tracheal injury was unknown. Most iatrogenic tracheal injuries are found after extubation, and a diagnostic delay is associated with a higher mortality. However, a prompt diagnosis is often difficult because of their rarity and the lack of the specific findings. The subpleural emphysema found by exploratory thoracoscopy led to the diagnosis in the present case. The tracheal laceration penetrated the membranous wall but did not yet perforate into the thoracic cavity. Detection of the subpleural emphysema allowed for an immediate diagnosis and also contributed to the favorable outcome.

Although the tracheal injury had already existed at the time of the intubation, it manifested later on. This delayed onset may be explained by the subsequent migration of the DLT with the inflated tracheal cuff placed below the injured area, preventing a transient exacerbation of the tracheal laceration [4]. This malposition also caused a higher PIP with hypoxia, and properly repositioning the tube position resulted in an air leak into the mediastinum with subpleural emphysema.

As surgical indication for tracheal injury is composed of several factors, including a perforation into the thoracic cavity, longer lacerations than 2 cm, clinically deteriorating condition, esophageal prolapse into the tracheal lumen, and ventilatory leak [2, 3, 5]. On the other hand, conservative treatment is applicable incases with lesser lacerations, a stable respiratory status, and no evidence of esophageal injury [2–4, 6].

The present case also poses a novel perspective on the morphological classification of tracheal injuries. Because the current classification of a level II injury was defined only by a muscular wall injury without any mediastinal involvement, it contained a broad spectrum of etiologies of both a tracheal perforation and penetration. Her injury was detected during the stage of tracheal penetration limited to the mediastinum and not a perforation into the thoracic cavity. A tracheal perforation could immediately lead to mediastinitis with a catastrophic outcome, whereas a tracheal penetration could be managed conservatively in selected patients. A tracheal penetration is deemed a preceding status of a perforation and a favorable prognostic sign of a level II injury. However, careful postoperative observation of the extent of the subcutaneous and mediastinal emphysema is essential to determine the timing of emergency surgery to repair the tracheal injury even among those cases.

## Conclusion

An iatrogenic tracheal injury by a DLT intubation could initially manifest as visceral subpleural emphysema. Once subpleural emphysema is observed during surgery, a prompt diagnostic workup should be performed.

## Data Availability

Not applicable.

## Abbreviations

DLT: Double-lumen tube
PIP: Peak inspiratory pressure
CT: Computed tomography
